# Cells expressing PAX8 are the main source of homeostatic regeneration of adult mouse endometrial epithelium and give rise to serous endometrial carcinoma

**DOI:** 10.1242/dmm.047035

**Published:** 2020-10-30

**Authors:** Dah-Jiun Fu, Andrea J. De Micheli, Mallikarjun Bidarimath, Lora H. Ellenson, Benjamin D. Cosgrove, Andrea Flesken-Nikitin, Alexander Yu. Nikitin

**Affiliations:** 1 Department of Biomedical Sciences, Cornell University, Ithaca, NY 14853, USA; 2 Meinig School of Biomedical Engineering, Cornell University, Ithaca, NY 14853, USA; 3 Cornell Stem Cell Program, Cornell University, Ithaca, NY 14853, USA; 4 Memorial Sloan-Kettering Cancer Center, New York, NY 10065, USA

**Keywords:** Endometrial cancer, Mouse models, Single-cell transcriptome, Stem cells

## Abstract

Humans and mice have cyclical regeneration of the endometrial epithelium. It is expected that such regeneration is ensured by tissue stem cells, but their location and hierarchy remain debatable. A number of recent studies have suggested the presence of stem cells in the mouse endometrial epithelium. At the same time, it has been reported that this tissue can be regenerated by stem cells of stromal/mesenchymal or bone marrow cell origin. Here, we describe a single-cell transcriptomic atlas of the main cell types of the mouse uterus and epithelial subset transcriptome and evaluate the contribution of epithelial cells expressing the transcription factor PAX8 to the homeostatic regeneration and malignant transformation of adult endometrial epithelium. According to lineage tracing, PAX8^+^ epithelial cells are responsible for long-term maintenance of both luminal and glandular epithelium. Furthermore, multicolor tracing shows that individual glands and contiguous areas of luminal epithelium are formed by clonal cell expansion. Inactivation of the tumor suppressor genes *Trp53* and *Rb1* in PAX8^+^ cells, but not in FOXJ1^+^ cells, leads to the formation of neoplasms with features of serous endometrial carcinoma, one of the most aggressive types of human endometrial malignancies. Taken together, our results show that the progeny of single PAX8^+^ cells represents the main source of regeneration of the adult endometrial epithelium. They also provide direct experimental genetic evidence for the key roles of the P53 and RB pathways in the pathogenesis of serous endometrial carcinoma and suggest that PAX8^+^ cells represent the cell of origin of this neoplasm.

## Abstract (precis)

**Summary:** The study describes a single-cell transcriptomic atlas of the mouse uterus and shows that PAX8^+^ cells are responsible for long-term maintenance of endometrial epithelium and might represent the cell of origin of serous endometrial carcinoma.

## INTRODUCTION

During reproductive age, in humans and mice, the endometrial epithelia and stroma undergo cell loss and regrowth in a cyclical manner in response to steroid hormones. During ovulation, there is an increase in progesterone levels that results in differentiation of the endometrium, with subsequent turnover in the absence of embryo implantation. In humans, this turnover involves endometrial shedding manifested by menstruation. In mice, the turnover is accomplished by cell apoptosis and reabsorption. In both species, cyclical homeostatic regeneration of the endometrial epithelium is expected to be maintained by stem cells. However, the presence and location of such cells has been actively debated for the past several decades (reviewed by Gargett et al., 2016; Wang et al., 2020).

A number of recent studies have suggested the location of stem cells in either the glandular (Kaitu'u-Lino et al., 2010; Syed et al., 2020) or luminal (Chan and Gargett, 2006; Huang et al., 2012; Jin, 2019) compartments of the mouse endometrial epithelium. In humans, such cells are commonly thought to be located in the basalis segment of the endometrial glands (Nguyen et al., 2012; Patterson and Pru, 2013; Valentijn et al., 2013). However, according to a recent single-cell transcriptome study of secretory-phase human endometrium, cells showing characteristics of stem/progenitor cells are located in the upper region of the functionalis (Wu et al., 2018 preprint). At the same time, several studies have suggested that endometrial epithelium can be regenerated by stem cells of either stromal/mesenchymal (Huang et al., 2012; Patterson et al., 2013) or bone marrow cell origin (Bratincsak et al., 2007; Du and Taylor, 2007; Ikoma et al., 2009; Mints et al., 2008; Taylor, 2004).

By analogy with stem cells in other organs and tissues (Fu et al., 2018), aberrations in the mechanisms governing endometrial epithelial stem cells can lead to a number of pathological conditions, including cancer. Uterine cancer is the fourth most frequent malignancy and the sixth cause of cancer-related deaths in women in the USA (Siegel et al., 2020). Although the incidence and mortality rates of some cancers, such as lung and colorectal cancers, are declining, both are increasing for endometrial carcinoma (Felix et al., 2017; Siegel et al., 2020). Endometrioid and serous subtypes are the most common subtypes of endometrial carcinoma and are characterized by distinct genetic alterations, pathological phenotypes and clinical behavior. Serous endometrial carcinoma (SEC) is the second most common type of endometrial carcinoma, accounting for ∼10-15% of all cases. SEC often presents at a late stage, recurs even after aggressive, adjuvant therapy and is responsible for the majority of deaths associated with endometrial carcinoma.

Recent extensive integrated genomic analyses of endometrial carcinomas have provided important insights into the repertoire of molecular aberrations characteristic of this malignancy (Berger et al., 2018; Kandoth et al., 2013). According to The Cancer Genome Atlas (TCGA) data, 77% of endometrioid carcinomas contain mutations in *PTEN*, but few alterations in *TP53* (1.1%) and the RB pathway, such as upregulation of *CCNE1* (none), *CDKN2A* (3%), *E2F1* (2.2%), *CDK2* (none), *CDK4* (1.1%) and *CDK6* (1.1%), and deletion of *RB1* (2.2%). SECs have *TP53* mutations in 95% of cases (Berger et al., 2018; Kandoth et al., 2013). Additionally, as the second most common alteration, >70% of SECs have aberrations in the RB pathway, involving upregulation of *CCNE1* (35%), *CDKN2A* (18%), *E2F1* (18%), *CDK2* (17%), *CDK4* (7%) and *CDK6* (12%), and deletion of *RB1* (7%). Unfortunately, utilization of this information is compromised because the originating cell types have not been determined.

In the present report, we describe a single-cell transcriptomic atlas of the main cell types of the mouse uterus and an epithelial specific subset transcriptome and identify PAX8^+^ cells as the main contributor to homeostatic regeneration of the endometrial epithelium. We also show that conditional inactivation of *Trp53* and *Rb1* in PAX8^+^ endometrial epithelial cells of adult mice leads to neoplasms that recapitulate human SEC.

## RESULTS

**Fig. 1.**
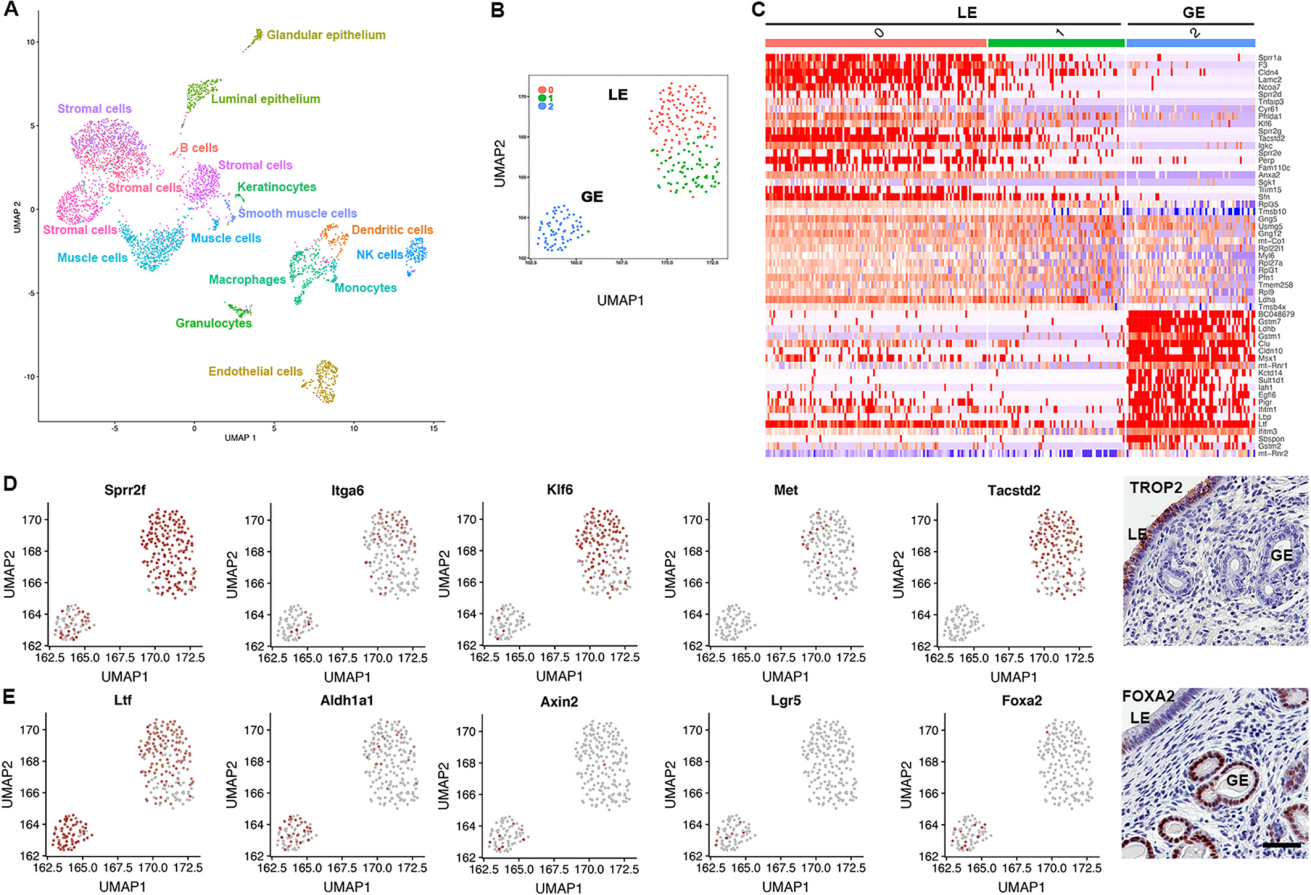
**Single-cell transcriptome analysis of the mouse uterus.** (A) UMAP projection of single-cell transcriptomic data from the mouse uterus, highlighting the main cell types. NK, natural killer. (B) UMAP projection of luminal (LE) and glandular (GE) epithelial populations labeled by group as identified by unsupervised SNN clustering. (C) Heatmap representing the top 55 differentially expressed genes across the three subpopulations identified in B and independent from prior labels (for the list of genes, see Table S1). (D,E) Preferential expression of genes in luminal (D) and glandular (E) clusters. Right panels, immunostaining for TROP2, encoded by *Tacstd2*, and FOXA2. ABC Elite, with Hematoxylin counterstaining. Scale bar: 50 µm (applicable to both images).

To identify cells with expression limited to endometrial epithelium, we have analyzed single-cell mRNA sequencing data published in the Mouse Cell Atlas (MCA; Han et al., 2018). The MCA contains >400,000 single-cell transcriptomic profiles from 51 mouse tissues, organs and cell cultures, including 3761 cells from the uterus. For the uterus, in-depth analysis of the data was not performed. However, two epithelial clusters were identified and annotated. One cluster was characterized by high *Ltf* expression, whereas the other had high expression of *Sprr2f*. To improve the resolution of these data, we have re-analyzed the MCA uterus dataset using the Seurat pipeline and sctransform data normalization method to account better for technical noise while preserving biological heterogeneity (Hafemeister and Satija, 2019 preprint). We then prepared a uniform manifold approximation and projection (UMAP) analysis of the main cell types of the mouse uterus (Fig. 1A) and an epithelial subset (Fig. 1B). Furthermore, we performed unsupervised shared nearest neighbor (SNN) clustering and revealed three subpopulations (Fig. 1C; Table S1). Two groups (0 and 1) had preferential expression of *Sprr2f* and *Tacstd2* (Fig. 1D)*.* Consistent with a previous report of luminal epithelium-specific *Tacstd2* expression (Filant and Spencer, 2013), TROP2, encoded by *Tacstd2*, has been detected exclusively in the luminal epithelium (Fig. 1D). Thus, groups 0 and 1 have been annotated as the luminal epithelium. Group 2 was characterized by preferential expression of *Ltf* and *Foxa2*. Based on glandular-specific expression of FOXA2 (Jin, 2019; Fig. 1D), this group was annotated as the glandular epithelium. Some markers associated with cell stemness and fate (*Itga6*, *Klf5*, *Met* and *Tacstd2*) were detected in the luminal subpopulations, whereas others (*Aldh1a1*, *Axin2*, *Lgr5* and *Foxa2)* were present in the glandular group (Fig. 1D,E). This suggests a possibility that the endometrium might contain not one but two stem cell pools.

**Fig. 2.**
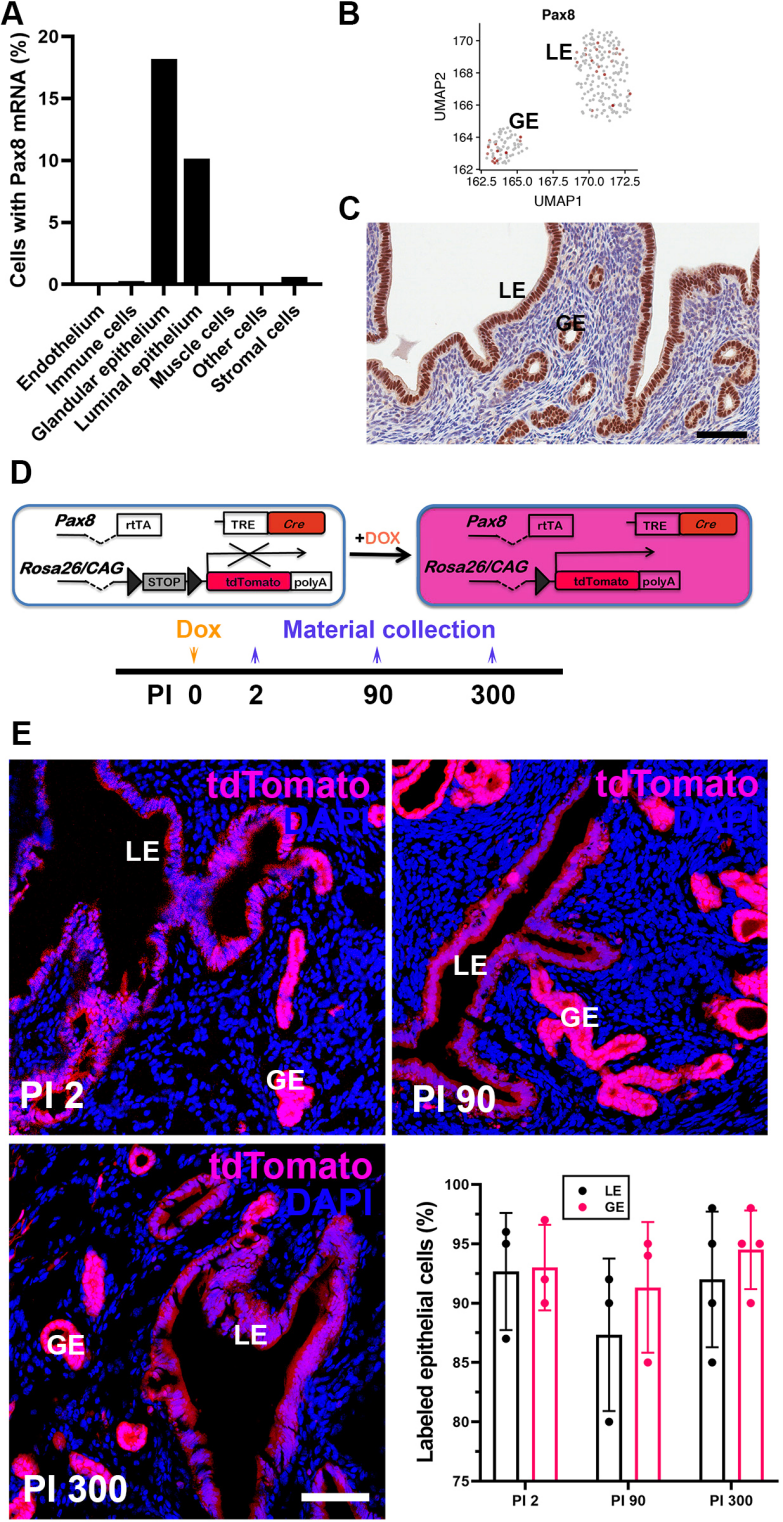
**Characterization of PAX8^+^ cells.** (A) Percentage of cells within the MCA with detected *Pax8* mRNA expression across uterine cell types. (B) Normalized *Pax8* mRNA expression in luminal (LE) and glandular (GE) epithelial cells. (C) Immunohistochemical detection of PAX8 expression in both luminal and glandular epithelial cells. ABC Elite, with Hematoxylin counterstaining. Scale bar: 50 µm. (D) Design for lineage tracing of PAX8^+^ cells. Dox, doxycycline; PI, days post-induction after a single doxycycline pulse. (E) Lineage tracing of PAX8^+^ cells in mouse endometrium collected at PI 2, 90 and 300. tdTomato expression, magenta; DAPI counterstaining, blue. Confocal microscopy. Scale bar: 75 µm. Quantification of labeled epithelial cells: PI 2, *n*=3; PI 90, *n*=3; PI 300, *n*=4. In all columns, data are the mean±s.d.; *P*-values are from Student's two-tailed unpaired *t*-test: *P*>0.05.

According to single-cell transcriptome analysis, expression of the transcription factor PAX8 was largely present in the endometrial epithelium (Fig. 2A), being detected in both luminal and glandular groups (Fig. 2B). Immunostaining showed the presence of PAX8 in epithelial but not stromal cells of the endometrium (Fig. 2C). To support this observation, we used Tg(Pax8-rtTA2S*M2)1Koes/J (Pax8-rtTA) Tg(tetO-Cre)1Jaw/J (Tre-Cre) mice (Perets et al., 2013) crossed to *Gt(ROSA)26Sor^tm9(CAG-tdTomato)Hze^*/J (Ai9) reporter mice (JAX stock no. 007909). In these mice, the *Pax8* promoter drives the expression of reverse transactivating protein (rtTA), which, in the presence of doxycycline, binds to the tetracycline response element (TRE), thereby leading to Cre-*loxP*-mediated activation of the reporter gene in Ai9 mice (Fig. 2D). In Ai9 mice, expression of red fluorescent protein variant (tdTomato) under the control of *CAG* promoter at the *Rosa26* locus is possible only after Cre-mediated deletion of the stop codon flanked by *loxP* sites (Madisen et al., 2010). Lineage tracing, using the Pax8-rtTA Tre-Cre Ai9 mice, revealed that >90% of endometrial epithelial cells were labeled 2 days after a single intraperitoneal administration of doxycycline (Fig. 2E). The majority of the luminal and glandular epithelium continued to express tdTomato for ≥300 days after the doxycycline pulse. These results support the notion that epithelial cells are responsible for long-term maintenance of the epithelium throughout the estrous cycle.

**Fig. 3.**
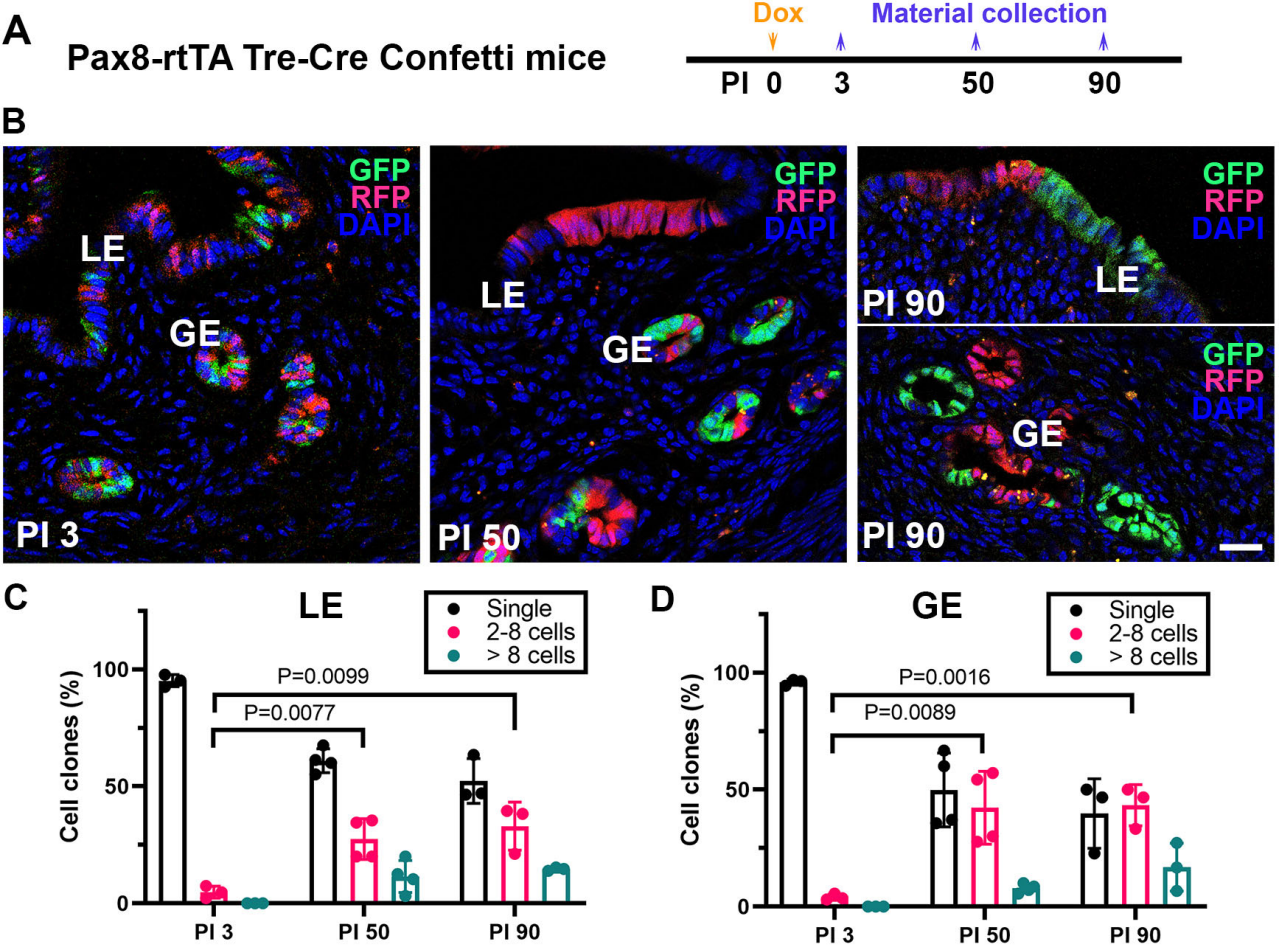
**Clonal expansion of PAX8-expressing cells in Pax8-rtTA Tre-Cre Confetti mice.** (A) Experimental design. Dox, doxycycline; PI, days post-induction after a single doxycycline pulse. (B) Detection of GFP (green) and RFP (red) at PI 3, 50 and 90. Note that a subset of PAX8^+^ cells has the ability to proliferate, resulting in large monochromatic clones. Counterstaining is with DAPI, blue. Confocal microscopy. Scale bar: 25 µm. (C,D) Quantification of clone size and distribution at PI 3, 50 and 90 in luminal (LE) and glandular (GE) epithelium. PI 3, *n*=3; PI 50, *n*=4; PI 300, *n*=3. All columns, data are the mean±s.d.; *P*-values are from Student's two-tailed unpaired *t*-test.

Using the Pax8-rtTA Tre-Cre model, but with Confetti mice [*Gt(ROSA)26Sor*^*tm1(CAG-Brainbow2.1)Cle*^/J; JAX stock no. 013731] instead of Ai9 reporter mice (Fig. 3A), we observed random labeling of individual cells with either green fluorescent protein (GFP) or red fluorescent protein (RFP) in both luminal and glandular epithelial compartments 3 days after a single doxycycline pulse (Fig. 3B-D). However, at later stages, both compartments became composed increasingly of monochromatic groups of cells (Fig. 3B-D). These findings show clonal expansion of PAX8-expressing endometrial epithelium.

**Fig. 4.**
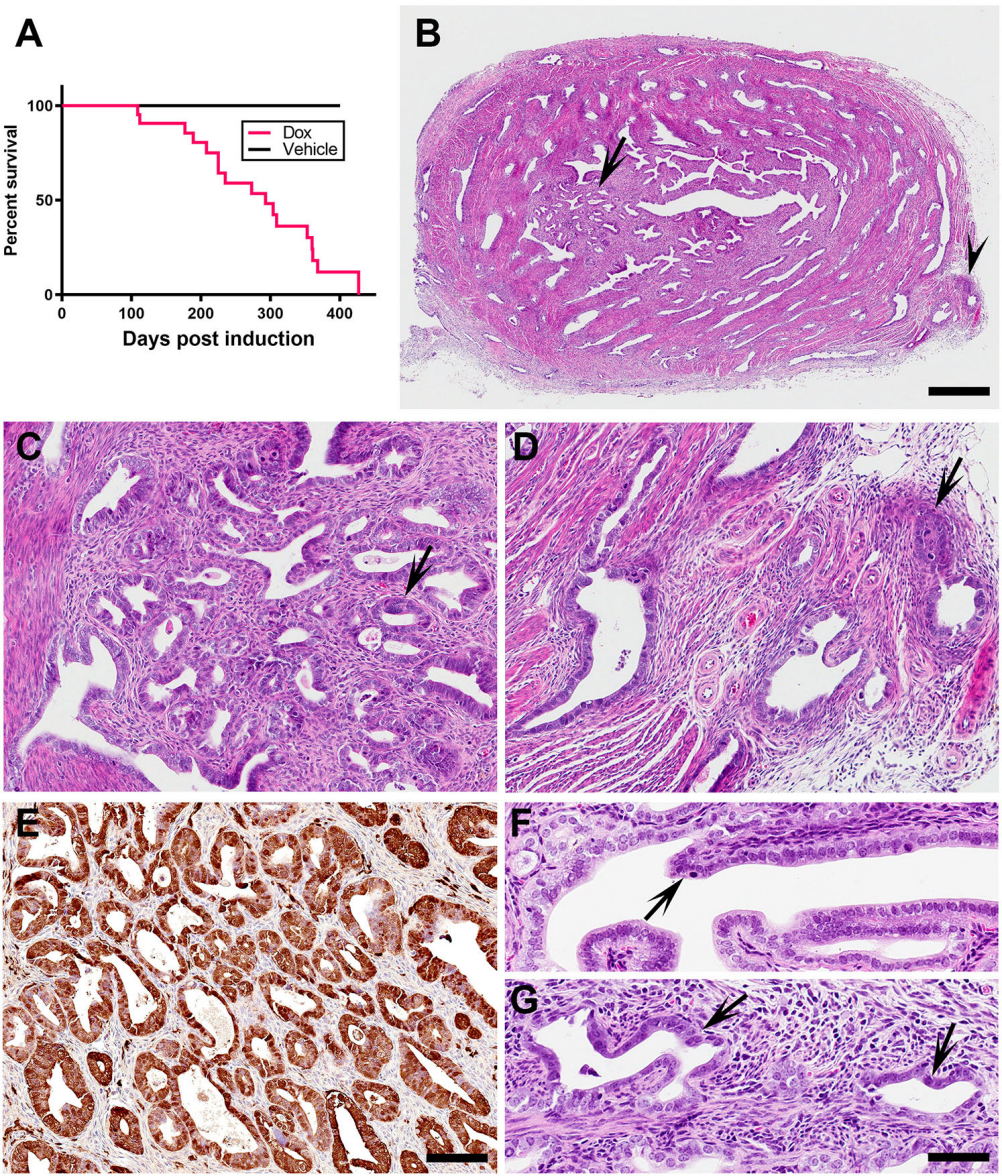
**Mouse model of SEC.** (A) Survival of Pax8-rtTA TRE-Cre *Trp53^loxP/loxP^ Rb1^loxP/loxP^* Ai9 mice that were treated with doxycycline (Dox, *n*=21) or vehicle (*n*=10); log-rank *P*=0.0004. (B-D) Cross-section of the uterus (B), with areas of neoplastic endometrial epithelium with marked cytological atypia and glandular pattern (C, arrow), and invasion into the myometrium and perimetrium (D, arrow). Arrow and arrowhead in B indicate the location of C and D, respectively. (E) tdTomato expression in the endometrial epithelial neoplasm developed 273 days after intraperitoneal doxycycline administration. (F,G) Dysplastic epithelial lesions (arrows) in luminal (F) and glandular (G) endometrial epithelium. (B-D,F,G) Hematoxylin and Eosin. (E) ABC Elite method, with Hematoxylin counterstaining. Scale bars: 600 µm in B; 100 µm in E (applicable to C and D); 50 µm in G (applicable to F).

Given that *TP53* mutations are present in 95% of SEC cases and alterations in the RB pathway in >70% of SEC cases, we conditionally inactivated *Trp53* and *Rb1* in the endometrial epithelium in adult (6- to 8-week-old) virgin Pax8-rtTA TRE-Cre *Trp53^loxP/loxP^ Rb1^loxP/loxP^* Ai9 mice. We observed formation of endometrial neoplasms in 81% (17 of 21) mice between 109 and 400 days after doxycycline administration (Fig. 4A). Histological characterization of uteri revealed invasion of malignant glands into the myometrium and serosa, marked cytological atypia and morphological similarity to human SEC (Fig. 4B-D). Supporting efficient Cre-*loxP*-mediated gene recombination, neoplastic cells expressed tdTomato (Fig. 4E). Beginning at 60 days post-injection, early dysplastic lesions characterized by loss of polarity, cellular atypia and cell proliferation were observed in both luminal (Fig. 4F) and glandular (Fig. 4G) endometrial epithelium.

By 300 days after doxycycline administration, 45% of mice developed dysplastic lesions in the epithelium of the uterine tube (also known as the oviduct or Fallopian tube; A.F.-N. and A.Y.N., unpublished observations.) Eighteen percent of the same mice developed more advanced but localized neoplasms. These lesions were similar to serous tubal intraepithelial carcinomas (STICs) and early high-grade serous carcinomas previously described in mouse models with combined inactivation of *Trp53* and *Brca1*, *Brca2* or *Pten* (Perets et al., 2013), or an amino-terminal truncated version of SV40 large T antigen (T121), which inactivates all members of the RB family (Rb1, p107 and p130) (Zhang et al., 2019). However, tubal lesions developed later than endometrial neoplasms, with no STICs being observed <154 days after doxycycline induction.

**Fig. 5.**
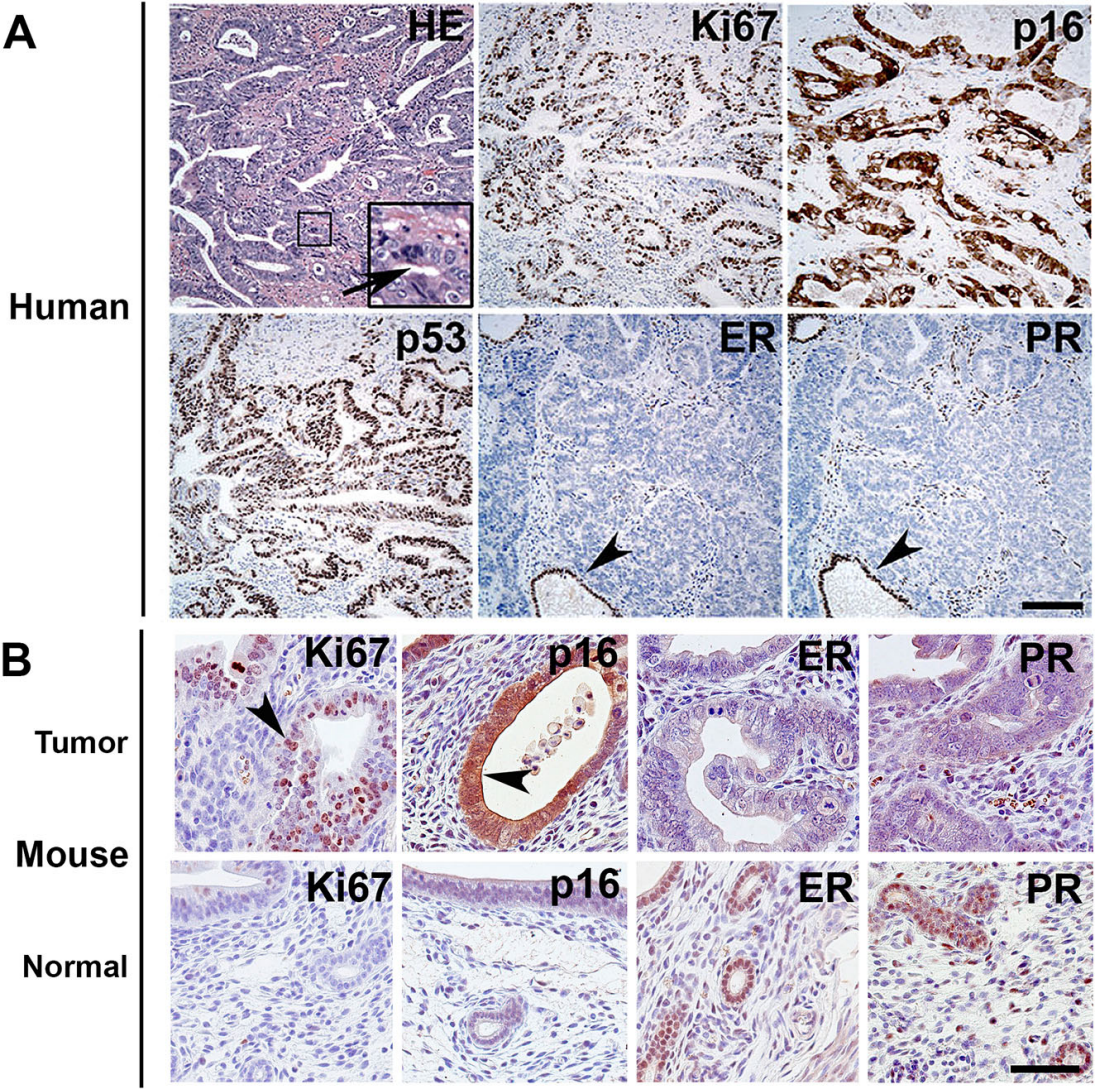
**Comparative immunophenotyping of SEC.** (A) Human SEC. Glandular pattern and marked cytological atypia (inset, arrow); intense, diffuse staining of p53, Ki67^+^ cells, lack of expression of estrogen receptor (ER); strong, diffuse staining of p16 and lack of progesterone receptor (PR) expression. Arrowheads, normal cells with expression of ER and PR near the tumor. HE, Hematoxylin and Eosin; other images, ABC Elite method, with Hematoxylin counterstaining. (B) Detection of the proliferation markers Ki67, p16, ER and PR in mouse endometrial tumor (top row) and normal tissue (bottom row). Arrowheads, Ki67^+^ cell and cytoplasmic staining of p16. ABC Elite method, with Hematoxylin counterstaining. Scale bars: 200 µm in A; 60 µm in B.

Human SECs are characterized by diffuse p53 staining, cytoplasmic p16 (also known as CDKN2A), reduced expression of estrogen receptor (ER; also known as ESR1) and progesterone receptor (PR; also known as NR3C3) and an increased number of Ki67^+^ (also known as MKI67^+^) cells (Fig. 5A). A similar pattern of p16, ER, PR and Ki67 expression has been observed in mouse endometrial neoplasms (Fig. 5B). Taken together with the histological features, this mouse model mimics many features of the human disease and is well suited for studying SEC.

**Fig. 6.**
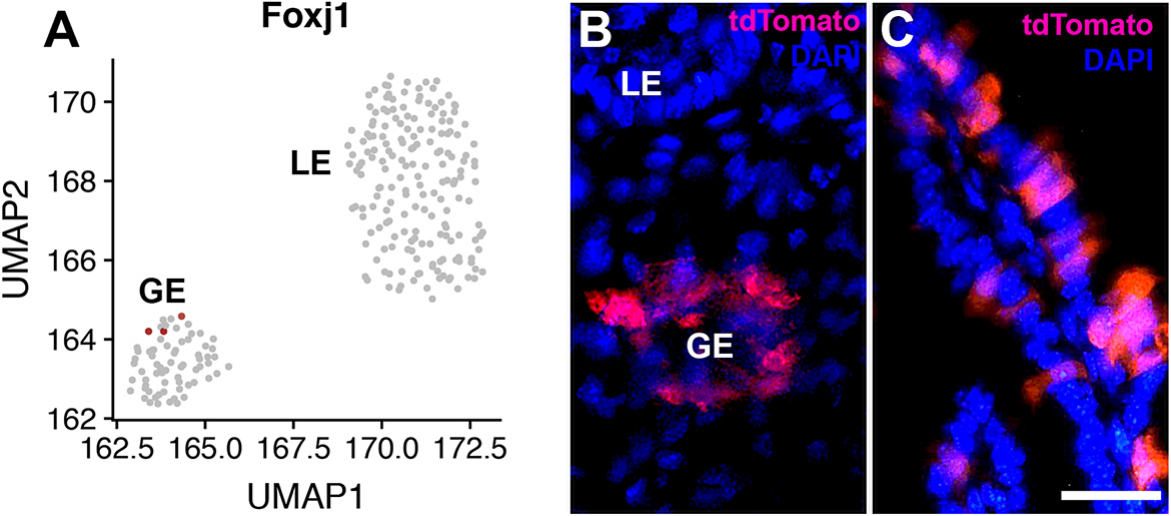
**FOXJ1 expression.** (A) Normalized *Foxj1* mRNA expression in epithelial cell subsets within the MCA. *FoxJ1* is detected in some endometrial glandular (GE) but not luminal (LE) epithelial cells. (B,C) tdTomato expression in GE (B) and uterine tube tubal epithelium (C) of FoxJ1^CreERT2::GFP^ Ai9 mouse at 1 day after intraperitoneal tamoxifen administration in the diestrous phase of the estrous cycle. Counterstaining with DAPI, blue. Scale bar: 50 μm.

To test whether PAX8^+^ cells were preferentially susceptible to *Trp53* and *Rb1* mutations, we inactivated these genes in FOXJ1^+^ ciliated endometrial epithelial cells using *Foxj1^tm1.1(cre/ERT2/GFP)Htg^*/J (FoxJ1^CreERT2::GFP^) mice. According to both mRNA levels and tdTomato expression in FoxJ1^CreERT2::GFP^ Ai9 mice, the frequency of FOXJ1^+^ cells was about one-third of that of PAX8^+^ glandular epithelial cells (Fig. 6A,B; M.B., A.F.-N. and A.Y.N, unpublished observations). However, none of the FoxJ1^CreERT2::GFP^ *Trp53^loxP/loxP^ Rb1^loxP/loxP^* Ai9 mice (*n*=14) developed any pathological lesions by 400 days after a single pulse of tamoxifen. In addition, neoplastic lesions were not observed in the epithelium of the uterine tube despite efficient labeling of tubal ciliated cells in our model (Fig. 6C).

## DISCUSSION

A mouse endometrial epithelium RNA-sequencing cell atlas during developmental stages has been reported previously (Wu et al., 2017). Of note, that study was focused only on CD326^+^ epithelial cells isolated by fluorescence-activated cell sorting and therefore did not allow identification of other epithelium-specific markers and non-epithelial cells. Our present analysis addresses these problems and provides a description of all main cell types of the mouse uterus.

Recently, two studies based on cell lineage tracing with tamoxifen-controlled Cre expression have been published (Jin, 2019; Syed et al., 2020). The first study used limited labeling and mathematical analysis in Krt19-Cre/ERT2 and FoxA2-Cre/ERT2 mice crossed with reporter mice (Jin, 2019) and concluded that the stem cell population resides in the intersection zone between luminal and glandular epithelium. By contrast, the second study reported that Axin2^+^ glandular cells can act as drivers of epithelial homeostasis and regeneration (Syed et al., 2020). Cell fate tracing was initiated at either diestrus (Jin, 2019) or metestrus (Syed et al., 2020), which complicates comparative interpretation of the results. Following of AXIN2^+^ cells was for periods ≤70 days for virgin mice. It has also been noted that AXIN2^+^ cells contribute to luminal epithelium only over long periods (Syed et al., 2020). Thus, the existence of alternative pools of luminal and/or glandular stem/progenitor cells cannot be excluded. This possibility is supported by our single-cell transcriptome findings suggesting that the endometrium might contain more than one stem cell pool.

Our studies involve the evaluation of the fate of PAX8-expressing cells. PAX8 is a member of the paired box (PAX) family of transcription factors. In adult mice, it is expressed in a number of tissues, including the epithelium of the uterine tube and the renal excretory system. In the endometrial epithelium, it is expressed in all stages of the estrous cycle (Syed et al., 2020). According to a single-cell transcriptome analysis, higher levels of *PAX8* mRNA are observed in the glandular epithelium than in the luminal epithelium. However, immunohistochemical analysis did not show any discernable differences in PAX8 expression. The mechanisms and functional significance of this observation remain to be studied.

Based on a single color tracing of PAX8^+^ cells (Syed et al., 2020) and analysis of cancer-associated mutations (Suda et al., 2018), recent studies have suggested that normal endometrial epithelial cells might expand clonally. Our findings based on two-color cell labeling provide direct support to this notion. It remains to be determined whether clonal expansion of the endometrial epithelium is a result of a neutral competition between endometrial epithelial stem cells, as has been shown for Lgr5^+^ cells in the small intestine (Snippert et al., 2010).

Our studies are consistent with the existence of endometrial epithelial stem cells. However, they cannot completely exclude non-epithelial contributions in non-homeostatic conditions, such as postpartum endometrial regeneration and artificial decidualization (Huang et al., 2012; Patterson et al., 2013). None of the studies reporting contributions of bone marrow-derived cells (Bratincsak et al., 2007; Du and Taylor, 2007; Ikoma et al., 2009; Mints et al., 2008; Taylor, 2004) included experiments testing for cell fusion, which is a common reason for the detection of bone-marrow-specific markers in non-hematopoietic tissues. Thus, these results await further confirmation.

The main strengths of autochthonous genetically modified mouse models of cancer include precise control of the initiation of genetic alterations and the ability to study cancer initiation and progression in an immunocompetent environment (Day et al., 2015; HogenEsch and Nikitin, 2012). Previously, it has been reported that endometrial epithelium-specific knockout of *Trp53* in Ksp1.3-Cre-expressing cells results in the development of endometrial intraepithelial carcinoma (the precursor of serous carcinoma) and serous carcinoma at 14-16 months of age (Wild et al., 2012). The use of this model for studies of adult endometrial carcinoma pathogenesis is complicated by constitutive expression of Ksp1.3-Cre in the embryonic precursor structures that give rise to most of the epithelia of the adult urinogenital system.

Our new model of SEC addresses the problem of constitutive expression and allows introduction of the initiating events in the adult mouse. It also confirms the crucial role of * if noe* mutations and RB1 pathway alterations in the pathogenesis of SEC. Thus, this model should be a valuable tool for future fundamental and translational studies. At the same time, our SEC model has two limitations and might require further refinements. First, we have noticed that in addition to development of uterine tube malignancies, ≤45% of mice form STICs and high-grade serous carcinomas in the uterine tube. These lesions develop later than the endometrial carcinomas, thereby reducing potential interference with the interpretation of our results. However, salpingectomy might allow establishment of a more accurate model of SEC. Given that most mice with serous uterine tube lesions also have SEC, it is possible that at least some of these lesions might represent metastasis from the neoplastic endometrial epithelium.

The second potential limitation is doxycycline-independent TRE-Cre activity in some cell types, such as thymic epithelial cells (Zhang et al., 2019) and blood cells (A.F.-N. and A.Y.N., unpublished observations). Depending on a combination of genetic alterations and mouse genetic backgrounds, TRE-Cre expression can lead to fast progression of background malignancies. As indicated in the Materials and Methods, ∼10% of TRE-Cre *Trp53^loxP/loxP^ Rb1^loxP/loxP^* mice developed histiocytic sarcomas and were not included in further analyses. Development of Pax8-Cre/ERT2 mice might be a better approach in future for modeling neoplastic lesions of the endometrial epithelium.

Our findings suggest that transformation of FOXJ1 ciliated cells might require other initiating genetic alterations. Indeed, it has been proposed that clear cell endometrial carcinomas can arise from ciliated endometrial cells (Cochrane et al., 2017). Compared with SECs, clear cell endometrial carcinomas have a lower frequency of *TP53* mutations (30-40% versus 95%) but contain other alterations, such as microsatellite instability (15% versus 5%) and *PTEN* mutations (30% versus 10%) (An et al., 2004; Lax et al., 1998).

## MATERIALS AND METHODS

### Experimental animals

The Tg(Pax8-rtTA2S*M2)1Koes/J (Pax8-rtTA) Tg(tetO-Cre)1Jaw/J (Tre-Cre) mice (Perets et al., 2013), *Foxj1^tm1.1(cre/ERT2/GFP)Htg^*/J (FoxJ1^CreERT2::GFP^; JAX stock no. 027012)**,** *Gt(ROSA)26Sor^tm9(CAG-tdTomato)Hze^* (Ai9) mice (JAX stock no. 007909) and Confetti mice [*Gt(ROSA)26Sor*^*tm1(CAG-Brainbow2.1)Cle*^/J; JAX stock no. 013731] were obtained from The Jackson Laboratory (Bar Harbor, ME, USA). The *Trp53^loxP/loxP^* and *Rb1^loxP/loxP^* mice, which have *Trp53* and *Rb1* genes, respectively, flanked by *loxP* alleles, were a gift from Dr Anton Berns (The Netherlands Cancer Institute, Amsterdam, The Netherlands). All the experiments and maintenance of the mice followed the recommendations of the Guide for the Institutional Laboratory Animal Use and Care Committee.

### Doxycycline and tamoxifen induction

For lineage tracing experiments, Pax8-rtTA Tre-Cre Ai9 and Pax8-rtTA Tre-Cre Confetti 6-week-old mice received a single dose (12 μl/g body weight) of doxycycline (6.7 mg/ml in sterile PBS) by intraperitoneal injection. For tumor induction experiments, doxycycline was administered to 6- to 10-week-old Pax8-rtTA Tre-Cre *Trp53*^*loxP/loxP*^ *Rb1*^*loxP/loxP*^ Ai9 mice and control mice. For tamoxifen induction of Cre expression in FoxJ1^CreERT2::GFP^ mice, 6-week-old mice received a single dose (8 μl/g body weight) of tamoxifen (25 mg/ml in corn oil; Sigma-Aldrich, St Louis, MO, USA; cat# T5648) by intraperitoneal injection. In our cohorts, ∼10% of TRE-Cre *Trp53^loxP/loxP^ Rb1^loxP/loxP^* mice developed histiocytic sarcomas and were not included in further analyses. All mice were euthanized by CO_2_ inhalation, and further analyses were carried out.

### Histology, immunohistochemistry and image analysis

All tissues were fixed in buffered 4% paraformaldehyde overnight at 4°C, followed by standard tissue processing and paraffin embedding. Histological and immunohistochemical staining was carried out on 4-μm-thick tissue sections. For immunohistochemistry, antigen retrieval was performed by incubation of deparaffinized and rehydrated tissue sections in boiling 10 mM sodium citrate buffer (pH 6.0) for 10 min. The primary antibodies against ER, FOXA2, Ki67, P16, PAX8, PR, tdTomato/RFP, TP53 and TROP2 were incubated at 4°C overnight, followed by incubation with secondary biotinylated antibodies [30 min, at room temperature (RT)]. A modified Elite avidin-biotin peroxidase (ABC) technique (Vector Laboratories, Burlingame, CA, USA; pk-6100) was performed at RT for 30 min. Hematoxylin was used as the counterstain. All primary antibodies used for immunostaining are listed in Table S2.

### Single-cell transcriptome analysis

The MCA (Han et al., 2018) expression matrices for uterus (Uterus1 GEO: GSM2906478; and Uterus2 GEO: GSM2906479) and related cell type metadata were downloaded (GEO: GSE108097) and re-analyzed using a custom version of the Seurat v.3.1.0 R package (Stuart et al., 2019). Seurat was used for data integration and normalization, principal component reduction, SNN clustering, UMAP visualization and differential gene expression analysis. We combined the Uterus1 and Uterus2 datasets and normalized the data using a regularized negative binomial regression model (sctransform R package) in order to remove technical effects while preserving biological variability (Hafemeister and Satija, 2019 preprint). On the normalized data, we performed SSN clustering with a resolution of 0.4, and we visualized the data using UMAP (Becht et al., 2018). Differential expression analysis was performed using the ‘FindAllMarkers’ function of Seurat, using Wilcoxon's signed-rank test and considering only genes with >log2(0.25) fold-change and expressed in ≥25% of cells in the cluster.

### Patient materials

All human specimens were de-identified. They were not collected specifically for the purpose of this research.

### Statistical analyses

Statistical comparisons were performed using Student's two-tailed unpaired *t*-test and a χ^2^ test with InStat 3 and Prism 8 software (GraphPad Software Inc., La Jolla, CA, USA). Survival curves were computed using the Kaplan–Meier method, and the survival comparisons were analyzed by log-rank tests. Significance was determined as *P*<0.05.

## Supplementary Material

Supplementary information

## References

[DMM047035C1] An H.-J., Logani S., Isacson C. and Ellenson L. H. (2004). Molecular characterization of uterine clear cell carcinoma. *Mod. Pathol.* 17, 530-537. 10.1038/modpathol.3800057 14976538

[DMM047035C2] Becht E., McInnes L., Healy J., Dutertre C. A., Kwok I. W. H., Ng L. G., Ginhoux F. and Newell E. W. (2018). Dimensionality reduction for visualizing single-cell data using UMAP. *Nat. Biotechnol.* 37, 38-44. 10.1038/nbt.4314 30531897

[DMM047035C3] Berger A. C., Korkut A., Kanchi R. S., Hegde A. M., Lenoir W., Liu W., Liu Y., Fan H., Shen H., Ravikumar V. et al. (2018). A comprehensive pan-cancer molecular study of gynecologic and breast cancers. *Cancer Cell* 33, 690-705.e9. 10.1016/j.ccell.2018.03.014 29622464 PMC5959730

[DMM047035C4] Bratincsak A., Brownstein M. J., Cassiani-Ingoni R., Pastorino S., Szalayova I., Tóth Z. E., Key S., Németh K., Pickel J. and Mezey E. (2007). CD45-positive blood cells give rise to uterine epithelial cells in mice. *Stem Cells* 25, 2820-2826. 10.1634/stemcells.2007-0301 17656643

[DMM047035C5] Chan R. W. S. and Gargett C. E. (2006). Identification of label-retaining cells in mouse endometrium. *Stem Cells* 24, 1529-1538. 10.1634/stemcells.2005-0411 16456137

[DMM047035C6] Cochrane D. R., Tessier-Cloutier B., Lawrence K. M., Nazeran T., Karnezis A. N., Salamanca C., Cheng A. S., McAlpine J. N., Hoang L. N., Gilks C. B. et al. (2017). Clear cell and endometrioid carcinomas: are their differences attributable to distinct cells of origin? *J. Pathol.* 243, 26-36. 10.1002/path.4934 28678427

[DMM047035C7] Day C.-P., Merlino G. and Van Dyke T. (2015). Preclinical mouse cancer models: a maze of opportunities and challenges. *Cell* 163, 39-53. 10.1016/j.cell.2015.08.068 26406370 PMC4583714

[DMM047035C8] Du H. and Taylor H. S. (2007). Contribution of bone marrow-derived stem cells to endometrium and endometriosis. *Stem Cells* 25, 2082-2086. 10.1634/stemcells.2006-0828 17464086

[DMM047035C9] Felix A. S., Yang H. P., Bell D. W. and Sherman M. E. (2017). Epidemiology of endometrial carcinoma: etiologic importance of hormonal and metabolic influences. *Adv. Exp. Med. Biol.* 943, 3-46. 10.1007/978-3-319-43139-0_1 27910063

[DMM047035C10] Filant J. and Spencer T. E. (2013). Cell-specific transcriptional profiling reveals candidate mechanisms regulating development and function of uterine epithelia in mice. *Biol. Reprod.* 89, 86 10.1095/biolreprod.113.111971 23946541 PMC7289334

[DMM047035C11] Fu D.-J., Miller A. D., Southard T. L., Flesken-Nikitin A., Ellenson L. H. and Nikitin A. Y. (2018). Stem cell pathology. *Annu. Rev. Pathol.* 13, 71-92. 10.1146/annurev-pathol-020117-043935 29059010 PMC5857951

[DMM047035C12] Gargett C. E., Schwab K. E. and Deane J. A. (2016). Endometrial stem/progenitor cells: the first 10 years. *Hum. Reprod. Update* 22, 137-163. 10.1093/humupd/dmv051 26552890 PMC4755439

[DMM047035C13] Hafemeister C. and Satija R. (2019). Normalization and variance stabilization of single-cell RNA-seq data using regularized negative binomial regression. *bioRxiv*. 10.1101/576827 PMC6927181 31870423

[DMM047035C14] Han X., Wang R., Zhou Y., Fei L., Sun H., Lai S., Saadatpour A., Zhou Z., Chen H., Ye F. et al. (2018). Mapping the mouse cell atlas by microwell-Seq. *Cell* 172, 1091-1107.e17. 10.1016/j.cell.2018.02.001 29474909

[DMM047035C15] HogenEsch H. and Nikitin A. Y. (2012). Challenges in pre-clinical testing of anti-cancer drugs in cell culture and in animal models. *J. Control. Release* 164, 183-186. 10.1016/j.jconrel.2012.02.031 22446384 PMC3387503

[DMM047035C16] Huang C.-C., Orvis G. D., Wang Y. and Behringer R. R. (2012). Stromal-to-epithelial transition during postpartum endometrial regeneration. *PLoS ONE* 7, e44285 10.1371/journal.pone.0044285 22970108 PMC3433810

[DMM047035C17] Ikoma T., Kyo S., Maida Y., Ozaki S., Takakura M., Nakao S. and Inoue M. (2009). Bone marrow-derived cells from male donors can compose endometrial glands in female transplant recipients. *Am. J. Obstet. Gynecol.* 201, 608.e1-608.e8. 10.1016/j.ajog.2009.07.026 19800602

[DMM047035C18] Jin S. (2019). Bipotent stem cells support the cyclical regeneration of endometrial epithelium of the murine uterus. *Proc. Natl. Acad. Sci. USA* 116, 6848-6857. 10.1073/pnas.1814597116 30872480 PMC6452687

[DMM047035C19] Kaitu'u-Lino T. J., Ye L. and Gargett C. E. (2010). Reepithelialization of the uterine surface arises from endometrial glands: evidence from a functional mouse model of breakdown and repair. *Endocrinology* 151, 3386-3395. 10.1210/en.2009-1334 20444944

[DMM047035C20] Kandoth C., Schultz N., Cherniack A. D., Akbani R., Liu Y., Shen H., Robertson A. G., Pashtan I., Shen R., Benz C. C. et al. (2013). Integrated genomic characterization of endometrial carcinoma. *Nature* 497, 67-73. 10.1038/nature12113 23636398 PMC3704730

[DMM047035C21] Lax S. F., Pizer E. S., Ronnett B. M. and Kurman R. J. (1998). Clear cell carcinoma of the endometrium is characterized by a distinctive profile of p53, Ki-67, estrogen, and progesterone receptor expression. *Hum. Pathol.* 29, 551-558. 10.1016/S0046-8177(98)80002-6 9635673

[DMM047035C22] Madisen L., Zwingman T. A., Sunkin S. M., Oh S. W., Zariwala H. A., Gu H., Ng L. L., Palmiter R. D., Hawrylycz M. J., Jones A. R. et al. (2010). A robust and high-throughput Cre reporting and characterization system for the whole mouse brain. *Nat. Neurosci.* 13, 133-140. 10.1038/nn.2467 20023653 PMC2840225

[DMM047035C23] Mints M., Jansson M., Sadeghi B., Westgren M., Uzunel M., Hassan M. and Palmblad J. (2008). Endometrial endothelial cells are derived from donor stem cells in a bone marrow transplant recipient. *Hum. Reprod.* 23, 139-143. 10.1093/humrep/dem342 17981818

[DMM047035C24] Nguyen H. P., Sprung C. N. and Gargett C. E. (2012). Differential expression of Wnt signaling molecules between pre- and postmenopausal endometrial epithelial cells suggests a population of putative epithelial stem/progenitor cells reside in the basalis layer. *Endocrinology* 153, 2870-2883. 10.1210/en.2011-1839 22474188 PMC3359601

[DMM047035C25] Patterson A. L. and Pru J. K. (2013). Long-term label retaining cells localize to distinct regions within the female reproductive epithelium. *Cell Cycle* 12, 2888-2898. 10.4161/cc.25917 24018418 PMC3899201

[DMM047035C26] Patterson A. L., Zhang L., Arango N. A., Teixeira J. and Pru J. K. (2013). Mesenchymal-to-epithelial transition contributes to endometrial regeneration following natural and artificial decidualization. *Stem Cells Dev.* 22, 964-974. 10.1089/scd.2012.0435 23216285 PMC3585744

[DMM047035C27] Perets R., Wyant G. A., Muto K. W., Bijron J. G., Poole B. B., Chin K. T., Chen J. Y., Ohman A. W., Stepule C. D., Kwak S. et al. (2013). Transformation of the fallopian tube secretory epithelium leads to high-grade serous ovarian cancer in Brca;Tp53;Pten models. *Cancer Cell* 24, 751-765. 10.1016/j.ccr.2013.10.013 24332043 PMC3917315

[DMM047035C28] Siegel R. L., Miller K. D. and Jemal A. (2020). Cancer statistics, 2020. *CA Cancer J. Clin.* 70, 7-30. 10.3322/caac.21590 31912902

[DMM047035C29] Snippert H. J., van der Flier L. G., Sato T., van Es J. H., van den Born M., Kroon-Veenboer C., Barker N., Klein A. M., van Rheenen J., Simons B. D. et al. (2010). Intestinal crypt homeostasis results from neutral competition between symmetrically dividing Lgr5 stem cells. *Cell* 143, 134-144. 10.1016/j.cell.2010.09.016 20887898

[DMM047035C30] Stuart T., Butler A., Hoffman P., Hafemeister C., Papalexi E., Mauck W. M. III, Hao Y., Stoeckius M., Smibert P. and Satija R. (2019). Comprehensive integration of single-cell data. *Cell* 177, 1888-1902.e21. 10.1016/j.cell.2019.05.031 31178118 PMC6687398

[DMM047035C31] Suda K., Nakaoka H., Yoshihara K., Ishiguro T., Tamura R., Mori Y., Yamawaki K., Adachi S., Takahashi T., Kase H. et al. (2018). Clonal expansion and diversification of cancer-associated mutations in endometriosis and normal endometrium. *Cell Rep* 24, 1777-1789. 10.1016/j.celrep.2018.07.037 30110635

[DMM047035C32] Syed S. M., Kumar M., Ghosh A., Tomasetig F., Ali A., Whan R. M., Alterman D. and Tanwar P. S. (2020). Endometrial Axin2+ cells drive epithelial homeostasis, regeneration, and cancer following oncogenic transformation. *Cell Stem Cell* 26, 64-80.e13. 10.1016/j.stem.2019.11.012 31883834

[DMM047035C33] Taylor H. S. (2004). Endometrial cells derived from donor stem cells in bone marrow transplant recipients. *JAMA* 292, 81-85. 10.1001/jama.292.1.81 15238594

[DMM047035C34] Valentijn A. J., Palial K., Al-Lamee H., Tempest N., Drury J., Von Zglinicki T., Saretzki G., Murray P., Gargett C. E. and Hapangama D. K. (2013). SSEA-1 isolates human endometrial basal glandular epithelial cells: phenotypic and functional characterization and implications in the pathogenesis of endometriosis. *Hum. Reprod.* 28, 2695-2708. 10.1093/humrep/det285 23847113

[DMM047035C35] Wang Y., Nicholes K. and Shih I. M. (2020). The origin and pathogenesis of endometriosis. *Annu. Rev. Pathol.* 15, 71-95. 10.1146/annurev-pathmechdis-012419-032654 31479615 PMC7980953

[DMM047035C36] Wild P. J., Ikenberg K., Fuchs T. J., Rechsteiner M., Georgiev S., Fankhauser N., Noske A., Roessle M., Caduff R., Dellas A. et al. (2012). p53 suppresses type II endometrial carcinomas in mice and governs endometrial tumour aggressiveness in humans. *EMBO Mol. Med.* 4, 808-824. 10.1002/emmm.201101063 22678923 PMC3494078

[DMM047035C37] Wu B., An C., Li Y., Yin Z., Gong L., Li Z., Liu Y., Heng B. C., Zhang D., Ouyang H. et al. (2017). Reconstructing lineage hierarchies of mouse uterus epithelial development using single-cell analysis. *Stem Cell Reports* 9, 381-396. 10.1016/j.stemcr.2017.05.022 28625536 PMC5511104

[DMM047035C38] Wu B., Li Y., Liu Y., Jin K., Zhao K., An C., Li Q., Gong L., Zhao W., Hu J. et al. (2018). Cell atlas of human uterus. *bioRxiv*. 10.1101/267849

[DMM047035C39] Zhang S., Dolgalev I., Zhang T., Ran H., Levine D. A. and Neel B. G. (2019). Both fallopian tube and ovarian surface epithelium are cells-of-origin for high-grade serous ovarian carcinoma. *Nat. Commun.* 10, 5367 10.1038/s41467-019-13116-2 31772167 PMC6879755

