# Correction: Cells expressing PAX8 are the main source of homeostatic regeneration of adult mouse endometrial epithelium and give rise to serous endometrial carcinoma

**DOI:** 10.1242/dmm.050537

**Published:** 2023-10-23

**Authors:** Dah-Jiun Fu, Andrea J. De Micheli, Blaine A. Harlan, Mallikarjun Bidarimath, Lora H. Ellenson, Benjamin D. Cosgrove, Andrea Flesken-Nikitin, Alexander Yu. Nikitin

**Affiliations:** ^1^ Department of Biomedical Sciences, Cornell University, Ithaca, NY 14853, USA; ^2^ Cornell Stem Cell Program, Cornell University, Ithaca, NY 14853, USA; ^3^ Meinig School of Biomedical Engineering, Cornell University, Ithaca, NY 14853, USA; ^4^ Memorial Sloan-Kettering Cancer Center, New York, NY 10065, USA

There was an error in *Dis. Model. Mech.* (2020) 13, dmm047035 (doi:10.1242/dmm.047035).

Blaine A. Harlan has now been included as an author of this article and author affiliations have been updated. The correct author list and affiliations are as shown above.

The Acknowledgements and Author contributions statement have also been corrected as below.

**Corrected:**

**Acknowledgements**

We thank Minseok (Joseph) Kim for his TROP2 immunostaining.

**Author contributions statement**

Conceptualization: B.A.H., L.H.E., B.D.C., A.F.-N., A.Y.N.; Methodology: D.-J.F., B.A.H., A.F.-N., A.Y.N.; Software: A.J.D., B.D.C.; Validation: D.-J.F., A.F.-N., A.Y.N.; Formal analysis: D.-J.F., A.J.D., A.F.-N., A.Y.N.; Investigation: D.-J.F., A.J.D., B.A.H., M.B., L.H.E., B.D.C., A.F.-N., A.Y.N.; Writing - original draft: A.F.-N., A.Y.N.; Writing - review & editing: D.-J.F., A.J.D., M.B., L.H.E., B.D.C., A.F.-N., A.Y.N.; Supervision: B.D.C., A.F.-N., A.Y.N.; Project administration: A.F.-N., A.Y.N.; Funding acquisition: B.D.C., A.F.-N., A.Y.N.

**Original:**

**Acknowledgements**

We thank Blaine A. Harlan for her technical contributions at the early stages of this project, and Minseok (Joseph) Kim for his TROP2 immunostaining.

**Author contributions statement**

Conceptualization: L.H.E., B.D.C., A.F.-N., A.Y.N.; Methodology: D.-J.F., A.F.-N., A.Y.N.; Software: A.J.D., B.D.C.; Validation: D.-J.F., A.F.-N., A.Y.N.; Formal analysis: D.-J.F., A.J.D., A.F.-N., A.Y.N.; Investigation: D.-J.F., A.J.D., M.B., L.H.E., B.D.C., A.F.-N., A.Y.N.; Writing - original draft: A.F.-N., A.Y.N.; Writing - review & editing: D.-J.F., A.J.D., M.B., L.H.E., B.D.C., A.F.-N., A.Y.N.; Supervision: B.D.C., A.F.-N., A.Y.N.; Project administration: A.F.-N., A.Y.N.; Funding acquisition: B.D.C., A.F.-N., A.Y.N.

Both the online full-text and PDF versions of the paper have been updated with these changes.

