# Supplementary material for: Cells expressing PAX8 are the main source of homeostatic regeneration of adult mouse endometrial epithelium and give rise to serous endometrial carcinoma
Source: Dis Model Mech. 2020 Oct 30;13(10):dmm047035. doi: 10.1242/dmm.047035 (PMC7648606; doi:10.1242/dmm.047035)
Supplement: Supplementary information [file dmm-13-047035-s1.pdf]

**Table S1. List of genes differentially expressed across three endometrial epithelial populations.**

Click here to Download Table S1

**Table S2. Primary antibodies used for immunostaining**

| Antigen | Antibody source, catalogue number | Clone | Dilution |
|---|---|---|---|
| ERα | Santa Cruz; sc-542 | F10 | 1:500 |
| FOXA2 | Abcam, ab108422 | EPR4466 | 1:100 |
| Ki67 | Dako, M7240 | K2 | 1:200 |
| P16 | Santa Cruz; sc-1207 | PC* | 1:100 |
| PAX8 | Proteintech Group; 10336-1-AP | PC | 1:100 |
| PR | Abcam, ab63605 | PC | 1:100 |
| TP53 | Thermo Scientific, MS-186-P | DO-7 | 1:1000 |
| tdTomato/RFP | Rockland Immunochemicals Inc., 600-401-379S | PC | 1:400 |
| TROP2 | Santa Cruz Biotechnology, sc-376181 | F-5 | 1:100 |

*PC, polyclonal

Disease Models & Mechanisms • Supplementary information
